# Epigenetic Modification: A Key Tool for Secondary Metabolite Production in Microorganisms

**DOI:** 10.3389/fmicb.2022.784109

**Published:** 2022-04-13

**Authors:** Sudha Bind, Sandhya Bind, A. K. Sharma, Preeti Chaturvedi

**Affiliations:** Department of Biological Sciences, CBSH, G. B. Pant University of Agriculture & Technology, Pantnagar, India

**Keywords:** secondary metabolites, epigenetics, chromatin remodeling, biosynthetic gene cluster, heritable changes

## Abstract

Microorganisms are stupendous source of secondary metabolites, having significant pharmaceutical and industrial importance. Genome mining has led to the detection of several cryptic metabolic pathways in the natural producer of secondary metabolites (SMs) such as actinobacteria and fungi. Production of these bioactive compounds in considerable amount is, however, somewhat challenging. This led to the search of using epigenetics as a key mechanism to alter the expression of genes that encode the SMs toward higher production in microorganisms. Epigenetics is defined as any heritable change without involving the changes in the underlying DNA sequences. Epigenetic modifications include chromatin remodeling by histone posttranslational modifications, DNA methylation, and RNA interference. Biosynthetic gene cluster for SMs remains in heterochromatin state in which the transcription of constitutive gene is regulated by epigenetic modification. Therefore, small-molecule epigenetic modifiers, which promote changes in the structure of chromatin, could control the expression of silent genes and may be rationally employed for discovery of novel bioactive compounds. This review article focuses on the types of epigenetic modifications and their impact on gene expression for enhancement of SM production in microorganisms.

## Introduction

Secondary metabolites (SMs) are low-molecular-weight compounds not directly involved in cell growth, development, or reproduction and produced when the active growth (cell expansion and cell division) stops. Transition from primary to secondary metabolism is achieved due to depletion of nutrients, absence of light, and change in the ambient pH (Yu and Keller, 2005). Microbes are prolific antibiotic factories (Awan et al., 2016) and proven to produce a variety of SMs that have been successfully used for drug development (Rutledge and Challis, 2015). Filamentous fungi are well known producers of secondary metabolites. Around 17,000 natural products have been identified and characterized from both marine and terrestrial organisms up to 2014 (Buckingham, 2015). More than 5,000 antibiotics have been produced from the actinobacteria to date and approximately 500 secondary metabolites isolated from myxobacteria (Challinor and Bode, 2015). *Streptomyces* is an efficient producer of 7,600 SMs identified until 2005 (Bérdy, 2005). These secondary metabolites have variable roles; some are beneficial while others are quite harmful for us. Fungal-derived beneficial secondary metabolites have been used as antibacterial, antifungal, antihypercholesterolemic, and immunosuppressants (Hoffmeister and Keller, 2007). However, some mycotoxins such as aflatoxins, gliotoxins, fusarins, and fumonisins are detrimental to human health (Wiemann and Keller, 2014). The presence of various SM gene clusters, revealed with the mining of microbial genome, suggested the possibility of augmentation of useful metabolites as well as checking the production of deleterious metabolites. Silent and cryptic pathway abundance in microbial genome also offers ample opportunity for new bioactive compound formation with high therapeutic properties.

**FIGURE 1:**
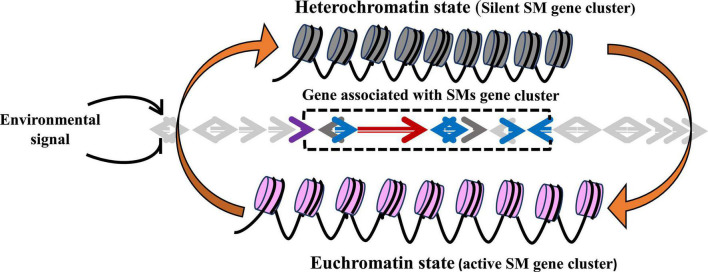
The impact of chromatin structure on transcriptional regulation of secondary metabolite (SM) gene clusters. SM gene clusters remain in transcriptionally inactive or repressive heterochromatin state. Due to different environmental cues (light, temperature, pH, carbon, nitrogen, and iron), chromatin changes from heterochromatin state to euchromatin (transcriptionally active) state, thus activating the SM biosynthetic pathway.

It is considered that genomics-based discovery of microbial SMs may be initiated by the genome sequencing of *S. coelicolor* and *S. avermitilis*, which led to the discovery of 22 (Bentley et al., 2002) and 25 (Omura et al., 2001) putative biosynthetic gene clusters (BGC), respectively, that could code for secondary metabolic pathways. Genome mining of *Aspergillus nidulans* revealed having 56 putative pathways (Yaegashi et al., 2014). All genes required for metabolite biosynthesis, regulation, and transport are located typically within the BGCs (Wisecaver et al., 2014). The onset of SM production typically occurs during early stationary phase and involves various metabolic changes among the microorganisms (Nieselt et al., 2010). The arrival of genomic era led to the discovery of various natural products from microorganisms. Studies of genome analysis of fungi revealed its tremendous potential for production of SMs. Under normal conditions, limited production of molecules has been observed from these microorganisms, which might be due to unexpressed genes. Hence, epigenetic modifiers have a potential role in the activation of silent genes and could possibly result in enhancing the production of natural metabolites (Figure 1). These epigenetic modifiers enhanced the production of SM by overexpressing the activator or repressor or by deletion of some genes (Mathur and Hoskins, 2017).

Most of the SMs are subjected to various complex regulatory systems comprising signaling cascade and pathway-specific regulators. Key metabolic enzymes such as polyketide synthase (PKS), terpene cyclase (TC), non-ribosomal peptide synthetase (NRPS), and dimethylallyl tryptophan synthetase (DMATS) are involved in the biosynthesis of huge number of secondary metabolites. However, few secondary metabolites are formed with other pathways; for example, oxylipins are derived from fatty acids. Biosynthetic genes of SMs are co-expressed and remain co-localized in gene cluster. Due to tight transcriptional regulation, most of these SM gene clusters remain silent under laboratory conditions. Activation of gene clusters has the potential for production of new natural products with high therapeutic leads. Various strategies were applied for activation of silent SM gene cluster including changes in the culture conditions (Bode et al., 2002) or co-cultivation of microbes. Most of the strategies were based on genetic manipulation such as overexpression of genes for pathway-specific transcription factor and key enzyme (Rosler et al., 2016) or manipulation of global regulators (Studt et al., 2016). Epigenetic research have shown that cells can undergo heritable changes in gene expression without changing the underlying DNA sequences. Secondary metabolite production by epigenetic modification has evolved as a powerful tool nowadays. Secondary metabolism in fungi and bacteria is controlled by elaborate regulatory network, which is influenced by various transcription factors and epigenetic modifiers (Cichewicz, 2010). By using the inhibitory compounds or gene knockout, the functionality or expression of regulatory enzymes present in SM biosynthetic pathway can be modified, and new natural products can be produced through epigenetic means.

### Production of SMs by Epigenetic Modifications

Epigenetics is an efficient tool for increased production of SMs. Epigenetic modification includes DNA methylation, chromatin remodeling, and RNA interference. These processes are vital for normal development and differentiation of cell and can be modified exogenously or by environmental factors. DNA methylation is a long-term epigenetic modification, whereas histone modification is a short-term or more flexible epigenetic modification. Epigenetic posttranslational modifications of histones include methylation, acetylation, ubiquitination of lysine, methylation of arginine, and phosphorylation of serine. DNA methylation and chromatin structure modulation play a major role in the overexpression of genes for SM production. This gives the opportunity to use epigenetic modifier compounds to induce the transcription of gene cluster (Yang et al., 2013). Histone deacetylase (HDAC) inactivation is considered as a good strategy for silent gene activation for secondary metabolite pathway and production of various bioactive compounds in filamentous fungi. *Aspergillus* is a large group of filamentous fungi with high capacity to produce secondary metabolites like penicillin, sterigmatocystin, aflatoxin, etc. Studies on penicillin and sterigmatocystin gene in *A. nidulans* by Kato et al. (2003) gave the understanding of secondary metabolite gene regulation. This study provides the guidance for similar kinds of studies in important species such as *A. flavus*, *A. parasiticus*, *Fusarium graminearum*, and *Penicillium chrysogenum* (Kosalkova et al., 2009; Payne and Yu, 2010). Recently, the role of chromatin structure and nucleosome modification in secondary metabolite gene expression has been reported from the studies in the model fungus *A. nidulans*. The first report of chromatin contribution was obtained after using HDAC deletion mutants in *A. nidulans* only. The inactivation of HDAC leads to the upregulation of secondary metabolite gene for enhanced production of sterigmatocystin, penicillin, and terrequinone A (Shwab et al., 2007).

### DNA Methylation

DNA methylation is important for normal development and differentiation of animals, plants, and microbial cells (Weber et al., 2007). DNA methylation plays a key role in the regulation of gene expression. DNA methylation of transposon and other highly repetitive DNA leads to the suppression of transposon expression and transposition and thus considered as a genome defense mechanism (Seidl and Thomma, 2017).

**FIGURE 2:**
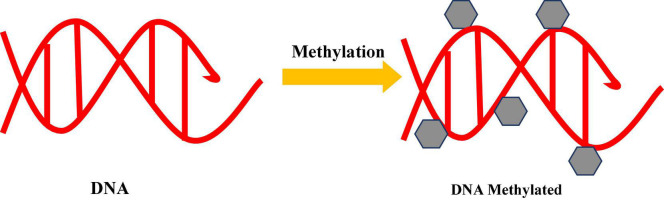
Epigenetic modulation showing DNA methylation: attachment of methyl group at 5′-carbon atom of cytosine ring.

DNA methylation includes covalent attachment of methyl group to the 5-carbon site of cytosine ring resulting in the formation of 5-methylcytosine that extends into the major groove of the DNA resulting in transcription inhibition. In the gene promoter region, hypermethylation is related to repression and hypomethylation is associated with activation of gene expression. The enzyme DNA methyltransferases (DNMTs) is responsible for methylation of cytosine 5′-carbon atom (Figure 2). In general, DNA methylation hinders or inhibits the transcription of machinery 5′ gene regulatory region. DNA methylation contains complexes of enzymes that are involved in histone modification, changing the chromatin structure and thus leading to activation and repression of transcription.

In fungi, the presence and importance of DNA methylation are still not clear. The absence of DNA methylation in some fungi has been observed using some highly sensitive techniques (Tang et al., 2012). DNA methylation has been reported in *Neurospora*, *Ascobolus*, and in other filamentous fungi. Chen et al. (2014) reported that *Tuber melanospermum* (truffle), having a methyl group attached to the 5’-carbon of cytosine ring (5 mC) at transposable element, accounts for 58% of genomic DNA. Decitabine (5-aza-20-deoxycytidine) and 5-azacytidine are frequently used as inhibitors of DNMT

**FIGURE 3:**
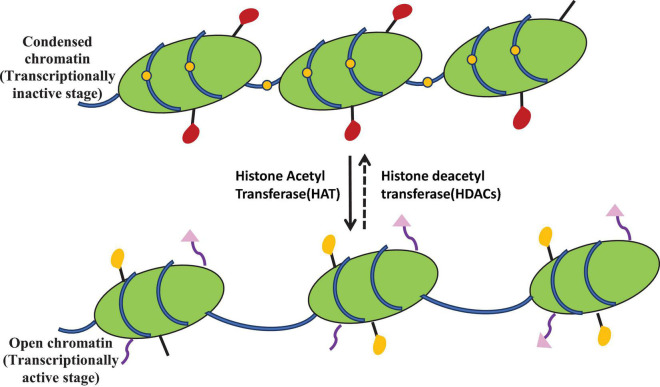
Inhibition of DNA methyltransferase (DNMTs) by using DNMT inhibitors. Azacytidine used as a DNMT inhibitor are base analogs that prevent the transfer of the methyl group to cytosine by incorporating into nucleic acid. Inhibitors covalently attach to nucleic acid and lead to a hypomethylated state and activation of repressed gene.

These drugs represent the analog of cytidine, having nitrogen atom at the 5-carbon instead of carbon in the pyrimidine ring. These molecules, by incorporating in DNA, prevent the proper transfer of methyl group by DNA methyltransferases. DNMT remain bounded to DNA in the presence of DNMT inhibitors, followed by destruction of DNMT by proteasome pathway. By using epigenetic modifiers, various secondary metabolites have been produced like tryptophan derivatives, namely, cytosporone, indigotide, and tenuipyrone in *Torrubiella luteorostrata* (Asai et al., 2011). A new aromatic polyketide glycoside, indigotide B, is produced from *Cordyceps indigotica* (Asai et al., 2012). Similarly, 5-azactytidine (5-AZA) is used as methyl transferase inhibitor. The interaction of 5-AZA with methyltransferase (Figure 3) responsible for DNA hypomethylation leads to chromatin restructuring (Fisch et al., 2009). A new secondary metabolite is produced in *Penicillium citreonigrum* by using AZA. The addition of epigenetic agent like AZA in culture medium containing nutrient broth, cornmeal, oatmeal, rice, and vermiculite leads to the production of metabolites such as sclerotiorin, sclerotiorimine, ochrephilone, dechloroisochromophilone III, dechloroisochromophilone IV, atlantinone A, and atlantinone B (Wang et al., 2010). The addition of two epigenetic modifiers suberohydroxamic acid (SBHA) and RG-108 in culture medium of *Isaria tenuipes* produced tenuipyrone (Asai et al., 2012). In *A. flavus* and *A*. *parasiticus*, there is a reduction of aflatoxin production after application of a DNA methylation inhibitor (Yang K. et al., 2014). According to Yang X. et al. (2014) and Zutz et al. (2013), there is a reduction of secondary metabolite production in endophyte *Pestalotiopsis crassiuscula* and in *A. clavatus*, respectively. Whereas, secondary metabolite increased in other fungi. Cultivation of marine fungus *Cochliobolus lunatus* (TA26–46) in the presence of 5-AZA produced seven new diethylene glycol phthalate ester monomers and oligomers, with four known analogs (Chen et al., 2016). The addition of 5-AZA and suberoylanilide hydroxamic acid (SAHA) in culture medium of marine fungus *Aspergillus* sp. (SCIOW2 strain) produced three new eremophilane-type sesquiterpenes (dihydrobipolaroxin B, C, and D) and a new dihydrobipolaroxin analog. The addition of nicotinamide or sodium butyrate (NaBut) in the culture medium of *Penicillium brevicompactum* resulted in a 2–10-fold increase in the phenolic compound production, some of which possessed cytotoxicity against liver carcinoma cells (El-Hawary et al., 2018). Table 1 presents the effect of exposure of microorganisms to epigenetic drugs. *P. crassiuscula*, an endophytic fungus isolated from leaves of *Fragaria chiloensis*, produced one new coumarin after supplementation of culture medium with 5-AZA (Yang K. et al., 2014). Coumarin, having various therapeutic properties, can be used as anticoagulant, anti-HIV, analgesic, antimicrobial, anti-inflammatory, and antineoplastic and has recently been reviewed as an antioxidant and immunomodulatory compound Sarker and Nahar (2017). Two DNA methyltransferases have been found in fungi with respect to enzymes involved; DIM-2 is found in *Neurospora crassa* (Kouzminova and Selker, 2001) and Masc2 in *Ascobolus immersus* (Chernov et al., 1997), which are involved in DNA methylation. DNA methylation with DIM-2 requires complex formation with heterochromatin protein 1 (HP1), the ortholog of *Schizosaccharomyces pombe SWI6* (Honda et al., 2012). A DNA methyltransferase, Masc 2 of *A. immerses*, plays a key role in the development of pre-meiotically induced DNA methylation during the sexual stage (Malagnac et al., 1997). RID ortholog of DNA methyltransferase in *N. crassa* is required for repeat-induced point (RIP) mutation during the sexual phase (Freitag et al., 2002). DNA methylation is not detected in *A. nidulans* and other *Aspergillus* spp. (Montanini et al., 2014), while RID and Masc1 (DmtA) detected in *A. nidulans* played an essential role in sexual development. The absence of DIM-2 and RID in the genome of *Schizosaccharomyces pombe* and *S. cerevisiae* (Proffitt et al., 1984) suggests lack of DNA methylation in these microbes. DNA methylation is rather stable, but for epigenetic modulation, the methyl group has to be removed. This can be done with active and passive processes or by a combination of both. Passive DNA methylation occurs on newly synthesized DNA strands during DNA replication rounds. Active methylation represents the oxidative modification of cytosine by 2-oxoglutarate-dependent cytosine dioxygenase enzymes of the ten-eleven translocation (TET) family. Oxidized cytosine can be removed completely during replication or may undergo enzymatic degradation (Wu and Zhang, 2011).

**TABLE 1:** The effect of exposure of microorganisms to epigenetic drugs.

Microorganisms	Epigenetic modifier	Induced compounds	References
*Diatrype* sp.	5-azacytidine	Lunalides A and B	Williams et al., 2008
*Cladosporium cladosporioides*	5-azacytidine and suberoylanilide hydroxamic acid	Cladochromes A, B, D–F, and G and calphostin B	Williams et al., 2008
*Aspergillus niger*	Suberoylanilide hydroxamic acid	Nigerone A	Henrikson et al., 2009
*Penicillium citreonigrum*	5-azacytidine	Sclerotiorin, sclerotioramine, dechloroisochromophilone III, dechloroisochromophilone IV, ochrephilone, 6-(3E, 5E)-5,7-dimethyl-2-methylenenona-3,5-dienyl)-2,4-dihydroxy-3- methylbenzaldehyde, and atlantinones A and B	Wang et al., 2010
*Alternaria* sp.	5-AZA and SAHA	Alternariol, alternariol-5-O-methyl ether, 30-hydroxyalternariol-5-O-methyl ether, altenusin, tenuazonic acid, and altertoxin II	Sun et al., 2012
*Hypoxylon* sp. CI-4	5-AZA and SAHA	VOCs, terpenes, primary and secondary alkanes, alkenes, organic acids, and benzene derivatives	Ul-Hassan et al., 2012
*Isariatenuipes*	SBHA and RG-108	Tenuipyrone	Asai et al., 2012
*Fusarium oxysporum* f. sp. *conglutinans*	Suberohydroxamic acid	5-butyl-6-oxo-1,6-dihydropyridine-2-carboxylic acid and 5-(but-9-enyl)-6-oxo-1,6-dihydropyridine-2-carboxylic acid	Chen et al., 2013
*Chaetomium indicum*	SBHA	Production of structurally diverse chaetophenol and some new polycyclic skeletons	Asai et al., 2013
*Pestalotiopsis crassiuscula*	5-azacytidine	4,6-dihydroxy-7-hydroxymethyl-3-methoxymethylcoumarin	Yang X. et al., 2014
*Chaetomium cancroideum*	Nicotinamide	Chaetophenol G and cancrolides A and B	Asai et al., 2015
*Aspergillus* sp.	5-AZA and SAHA	Production of three new eremophilane-type sesquiterpenes, (dihydrobipolaroxin B, C, and D) and new dihydrobipolaroxin analogue	Wang et al., 2016
*Chaetomium* sp.	Suberoylanilide hydroxamic acid or 5-azacytidine	Isosulochrin	Akone et al., 2016
*C. cancroideum*	Nicotinamide	Enhanced production of branched aliphatic and aromatic polyketides; production of new secondary metabolites (chaetophenol G and cancrolides A and B)	Asai et al., 2016
*Muscodor yucatanensis* Ni30	5-AZA, SAHA	Enhanced production of VOCs, ergosterol, and xylaguaianol C	Qadri et al., 2016
*Aspergillus* sp. SCSIOW3	SBHA and 5-AZA	Production of a new diphenylether-O-glycoside (1, diorcinol 3-O-a-D-ribofuranoside)	Li X. et al., 2017
*Phoma* sp. nov. LG0217	Suberoylanilide hydroxamic acid	(10’S)-verruculide B and vermistatin dihydrovermistatin	Gubiani et al., 2017
*Aspergillus versicolor*	SAHA	Production of 2,4-dimethoxyphenol and diorcinol and a new biphenyl derivative versiperol A	Zhu et al., 2018
*Lachnum palmae*	SAHA	Production of 18 dihydroisocoumarins, including five unknown brominated and two chlorinated compounds	Zhao et al., 2018
*Penicillium brevicompactum*	Nicotinamide	p-anisic acid, benzyl anisate, syringic acid, sinapic acid, acetosyringone, phenyl acetic acid, gentisaldehyde, and p-hydroxy benzaldehyde	El-Hawary et al., 2018
*Phomopsis heveicola*	Valproic acid	Cyclopentadecanolide, oxathiazole 2 thione, and pyrrolo[1,2-a]pyrazine-1,4-dione, hexahydro-3-(2 methyl), methyl 2,3-anhydro-4-6-O-benzylidenehexopyranoside	Ameen et al., 2021

*(5-AZA- 5-azacytidine; SAHA- suberoylanilide hydroxamic acid; SBHA- suberohydroxamic acid).*

### Chromatin Remodeling

Chromatin is a complex of chromosomal DNA and protein present inside the nucleus of eukaryotic cell. In the chromatin formation, highly condensed DNA wraps around the histone proteins to form nucleosome. Nucleosome is formed of 146 base pairs of DNA and eight histone proteins. In response to physiological and developmental signals, histones create variations in packaging and accessibility of DNA (Cedar and Bergman, 2009). To control the SM production, many chromatin modifiers have been discovered in filamentous fungi (Gacek and Strauss, 2012).

Chromatin modification is mediated through posttranslational modifications including methylation and acetylation of histone tail, thus leading to regulation of gene expression (Marks et al., 2001). At sub-telomeric region, genes for SMs are clustered and co-regulated, and at these sites, histone methylation and acetylation have major impact on the transcription (Yu and Keller, 2005; Gacek and Strauss, 2012). Several biosynthetic pathways of secondary metabolite-specific transcription factors are also embedded inside the gene cluster, namely, *aflR* regulating the synthesis of aflatoxin in *A. flavus* and *A. parasiticus* (Smith et al., 2007) and sterigmatocystin (ST) production in *A. nidulans* (Hoffmeister and Keller, 2007). Activators like aurofusarin and trichotecene clusters are also present inside the biosynthetic gene cluster of *F. graminearum* (Malz et al., 2005). According to Shwab et al. (2007) and Fisch et al. (2009), the fungal biosynthetic gene cluster situated at the distal end of the chromosome remains in the heterochromatin state, where genes are controlled by epigenetic modification like histone methylation and histone acetylation. Histone modification through acetylation and methylation has significant effects on specific fungal metabolite production (Bok et al., 2009). Histone deacetylase and methyltransferase are important tools in tracking of biosynthetic silent pathway and activating the silent gene cluster (Asai et al., 2012). Epigenetic agents have been used for alteration of transcription rate of some genes, thus significantly inducing the gene expression that is essential in secondary metabolite production (Henrikson et al., 2009).

**FIGURE 4:**
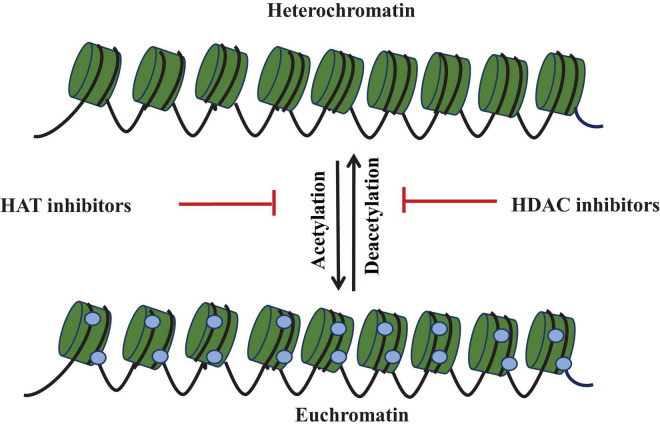
Epigenetic modulation showing histone acetylation mediated by histone acetyltransferase (HAT) and deacetylation mediated by histone deacetylase (HDAC). HDAC inhibitors activate the silent gene by inhibiting the histone deacetylase. These labels are associated with chromatin modulation required for cryptic gene expression.

Histone acetylation catalyzed by histone acetyltransferases causes reduction in DNA–histone interaction and thus leads to more open chromatin conformation and hence associated with altered gene expression (Javaid and Choi, 2017). For secondary metabolite activation, chromatin packaging has been influenced by the expression of artificial histone modification gene and by using inhibitors against histone deacetylase enzyme. Among various epigenetic regulators, HDAC plays a significant role and influences the DNA replication, transcription, and repair process (Robyr et al., 2002). HDACs remove the acetyl group from lysine residue of histone proteins (Figure 4). Several reports demonstrated the association of histone deacetylation with heterochromatic region and gene silencing (Bulger, 2005). HDAC modification led to the production of secondary metabolite in various fungi. Many new compounds are formed by using HDACs in the last two decades. On the basis of structure, HDAC inhibitors belong to four classes: cyclic peptides, hydroxamates, aliphatic acids, and benzamides. Most recent studies on filamentous fungi showed the frequent use of SAHA, trichostatin A (TSA), and NaBut as HDAC inhibitors. These HDAC inhibitors modify the gene expression patterns and encourage the change in non-histone protein at posttranslational level (Kim and Bae, 2011). TSA was firstly isolated from *Streptomyces hygroscopicus* strain and showed a specific inhibitory effect on HDACs *in vitro* and *in vivo*. SAHA and TSA belong to the hydroxamate group of HDAC inhibitors, bind to the Zn^++^ ion of HDAC active sites, and prevent their activity (Marks and Breslow, 2007). NaBut is a natural molecule that inhibits the histone deacetylase activity even at very low concentrations. The exact mechanism of NaBut action is not known, but it is reported that it attaches to the hydrophobic pocket of enzyme and performs as a non-competitive inhibitor of HDACs (Davie, 2003). Shwab et al. (2007) reported that deletion of hdaA gene encoding HDAC enzyme resulted in transcriptional activation of biosynthetic gene cluster of sterigmatocystin, penicillin, and terrequinone A from *A. nidulans* strain A89. Deletion of histone deacetylase (hdaA) in *A. fumigatus* increased the production of fumitremorgin B and pseurotin and decreased the gliotoxin production. In *A. fumigatus* strain AF293, transcription of NRPS gene cluster increased after deletion of homolog of hdaA (Lee et al., 2009); Mao et al. (2015) reported that deletion of hdaA activated secondary metabolite biosynthetic gene up to 75% and produced four new compounds in *Calcarisporium arbuscular*. In *Pestalotiopsis fici*, deletion of hdaA activated the production of various macrolides (Wu et al., 2016). Acetylation of histone H4 is related to the expression of toxin in *Aspergillus* species, and reduction in acetylation leads to the inhibition of toxin production (Roze et al., 2011). Histone deacetylase (hdaA) is used in the regulation of secondary metabolite production in *A. fumigatus*. Suppression of this gene causes the increased production of secondary metabolites and reduced gliotoxin production. However, the overexpression of hdaA increased the gliotoxin production (Lee et al., 2009). A new metabolite cytosporones A, B, and C increased after using HDAC inhibitors (Beau et al., 2012). Ding et al. (2020) reported that deletion of hdaA gene associated with histone deacetylase enzyme in *P. chrysogenum* induced the change in the secondary metabolite pathway and formed the indole alkaloid meleagrin. Li G. et al. (2017) studied that by using NAD^+^-dependent HDAC inhibitor nicotinamide, two new decalin-containing compounds, eupenicinicols C and D, and associated compounds like eujavanicol A and eupenicinicols A are produced from endophytic fungus *Eupenicillium*. The use of suberoylanilide hydroxamic acid, a histone deacetylase inhibitor, stimulated calphostin B and new cladochromes in *Cladosporium cladosporioides* (Williams et al., 2008). In *A*. *nidulans*, histone deacetylase inhibitors lead to overexpression of secondary metabolite genes (Reyes-Dominguez et al., 2007**).**

Besides histone acetylation, there are some other means of chromatin modification that regulate SM gene cluster expression (Strahl and Allis, 2000). Methylation of lysine is a complex modification and has an impact on activation and repression of gene transcription (Rolando et al., 2013). The addition of methyl group occurs on three different sites of lysine (mono-, di-, and tri-methylation) and at two sites of arginine residue (mono- and di-methylation). The number of methyl groups and their attachment site cause a different effect on chromatin structure and gene regulation. Methylation of lysine 9 activates the gene present inside the sterigmatocystin cluster. H3K9 methylation linked with heterochromatin protein A (HepA) is accountable for the formation of heterochromatin. The removal of HepA gene led to the *stc* gene cluster activation (Brakhage, 2013). SM regulator Lae A is indirectly involved in histone methylation by influencing H3K9 methylation. Lae A has been implicated in production of secondary metabolite in fungi, such as *Aspergillus terreus* (Palonen et al., 2017), *Penicillium expansum* (Kumar et al., 2018), *A. ochraceus* (Wang et al., 2019), *A. flavus* (Zhi et al., 2019), and *P. dipodomyis* (Yu et al., 2019). Loss of function of cclA gene, which is involved in histone H3 lysine 4 methylation, activated the secondary metabolite gene cluster expression and produced emodin, monodictyphenone, and its derivatives (Bok et al., 2009). Deletion of *Set1* gene, which codes for histone methyltransferase in *F. graminearum*, leads to the production of aurofusarin and deoxynivalenol (Liu et al., 2015). The deletion of *dot1* gene, which codes for H3K79 transferase in *A. flavus*, decreased aflatoxin production (Liang et al., 2017). The deletion of RmtA gene of arginine methyltransferase decreased the production of aflatoxin B1 (Satterlee et al., 2016). In *F. graminearum*, H3K27me3 is localized in sub-telomeric regions, and these regions were enriched of putative SM gene clusters.

In prokaryotes, histone is completely absent. However, bacterial genome, for instance *Streptomyces*, contains a form of HDAC and has a role in regulation of metabolism (Williams and Rimsky, 1997). A bifunctional nucleoid-associated protein (DdbA) has been characterized in *Streptomyces*, which consists of N terminal DNA-binding histone H1-like domain DksA at the C terminal end, having the ability to modulate the RNA polymerase to encourage the transcription (Aldridge et al., 2013).

### RNA Interference

Non-coding RNA (ncRNA) are functional RNA transcribed from DNA but not translated into protein and play an important role in the regulation of gene expression at transcriptional and posttranscriptional level. Non-coding RNA are divided into two categories, short ncRNAs and long ncRNAs. Short ncRNAs comprise microRNA (miRNA), short interfering RNAs (siRNA), and Piwi interacting RNAs (piRNA). miRNAs and siRNAs inhibit the translation by binding to the target messenger mRNA with complementary sequences leading to their degradation. All eukaryotic cells, including fungi, comprise long ncRNA (Donaldson and Saville, 2012). Some long ncRNAs are ribosome components (5.8S, 18S, and 26S rRNA). Natural antisense transcripts (NATs), a subset of long ncRNAs, have complementary sequences like other RNAs. NATs are divided into cis and trans NATs. NATs play a significant role in double-stranded RNA, chromatin remodeling, and transcriptional interference. SiRNA associate with Argonaute family protein, guide them toward RNA target, and regulate various gene expressions. Inhibition of specific genes in SM biosynthesis was carried out with RNA interference to control the production of various bioactive compounds. In *P. chrysogenum*, gene suppression leads to the production of methyltransferase that converts the meleagrin into glandiocoilin. Suppression of two other genes into same gene cluster leads to inhibition of roquefortine C and meleagrin, clearly suggesting that this cluster is responsible for the synthesis of both metabolites (García-Estrada et al., 2011). In another strain of *P. chrysogenum*, silencing of gene responsible for oxalate production leads to enhanced production of cephalosporin precursor, adipolyl-6 aminopenicillin acid. In *P. expansum*, inhibition of the gene responsible for toxin patulin production was carried out for reducing the toxicity of microorganism related with intake of patulin contaminant of orange juice. This gene inhibition is helpful in generation of two mutants with low production of patulin (Sanzani et al., 2012).

## Conclusion

The importance of microbial secondary metabolites in various industries aroused the interest in regulation of these metabolites by manipulating their pathway of synthesis. The richness of silent and cryptic pathways in genomes of microbes provides excellent opportunities for production of new natural compounds with high therapeutic properties. These regulatory pathways could be manipulated toward increased production of secondary metabolites. To achieve this, there is a challenge of activating the many silent gene clusters. Besides, it is also important to learn about how secondary metabolism is regulated and how gene clusters are activated or silenced. This review summarizes the different types of epigenetic regulation for increased production of SMs in microbes. Nowadays, epigenetics is an emerging tool increasingly gaining importance in microbial biotechnology for production of new bioactive compounds and their enhanced concentration in microorganisms. The soaring demand for novel drugs increased the requirement of alternative epigenetic modifiers on one hand and smart methods for high-throughput natural product discovery on the other. Epigenetic modifiers play a key role in the activation of silent gene cluster of SMs that has the ability to increase the production of various bioactive compounds. Epigenetic modification is quite challenging, yet it is one of the highly efficient tools used for the synthesis of industrial secondary metabolites having pharmaceutical and nutraceutical values. It is, however, very important to advance our knowledge about the effect of epigenetic modifiers on chromatin structure and their efficiency in activation of silent biosynthetic gene cluster in order to enhance the SM production.

## Future Prospects

Epigenetic modification is a key tool for gene expression and SM production. Despite the advance research, tools, and techniques in the field of molecular biology and biotechnology, there are some questions that are still unsolved with regard to epigenetic modification. Research is needed to focus on understanding the molecular mechanism of various factors involved in epigenetic modification so as to make the process more convenient and feasible at industrial scale. This will eventually revolutionize the research in microbial world for metabolite production.

## Author Contributions

All authors listed have made a substantial, direct, and intellectual contribution to the work, and approved it for publication.

## Conflict of Interest

The authors declare that the research was conducted in the absence of any commercial or financial relationships that could be construed as a potential conflict of interest.

## Publisher’s Note

All claims expressed in this article are solely those of the authors and do not necessarily represent those of their affiliated organizations, or those of the publisher, the editors and the reviewers. Any product that may be evaluated in this article, or claim that may be made by its manufacturer, is not guaranteed or endorsed by the publisher.
